# Study on Electrohydrodynamic Rayleigh-Taylor Instability with Heat and Mass Transfer

**DOI:** 10.1155/2014/485807

**Published:** 2014-01-06

**Authors:** Mukesh Kumar Awasthi, Vineet K. Srivastava

**Affiliations:** ^1^Department of Mathematics, College of Engineering, University of Petroleum and Energy Studies, Dehradun 248007, India; ^2^ISRO Telemetry, Tracking and Command Network (ISTRAC), Bangalore 560058, India

## Abstract

The linear analysis of Rayleigh-Taylor instability of the interface between two viscous and dielectric fluids in the presence of a tangential electric field has been carried out when there is heat and mass transfer across the interface. In our earlier work, the viscous potential flow analysis of Rayleigh-Taylor instability in presence of tangential electric field was studied. Here, we use another irrotational theory in which the discontinuities in the irrotational tangential velocity and shear stress are eliminated in the global energy balance. Stability criterion is given by critical value of applied electric field as well as critical wave number. Various graphs have been drawn to show the effect of various physical parameters such as electric field, heat transfer coefficient, and vapour fraction on the stability of the system. It has been observed that heat transfer and electric field both have stabilizing effect on the stability of the system.

## 1. Introduction

The potential flow of an incompressible fluid is a solution of the Navier-Stokes equation in which velocity **u** can be expressed as a gradient of potential function which satisfies Laplace's equation. The viscous potential flow (VPF) theory is also based on the assumption that velocity is given by the gradient of the potential function, but viscosity is nonvanishing. In this theory, the irrotational shearing stresses are assumed to be zero and viscosity comes through normal stress balance. The instability of the plane interface separating two fluids having different densities when the lighter fluid is accelerated toward the heavier fluid is called Rayleigh-Taylor instability. In 1999, Joseph et al. [1] studied the viscous potential flow analysis of Rayleigh-Taylor instability and observed that the wavelength of the most unstable wave increases strongly with viscosity. In 2002, Joseph et al. [2] extended their study of Rayleigh-Taylor instability to viscoelastic fluids at high Weber number (the ratio of the inertial force to the surface tension force) and concluded that the most unstable wave is a sensitive function of the retardation time, which fits into experimental data when the ratio of retardation time to that of relaxation time is of order 10^−3^.

In recent years, a great deal of interest has been focused on the study of heat and mass transfer on the stability of fluids flows because heat and mass transfer phenomenon is encountered in a wide variety of engineering applications such as boiling heat transfer and geophysical problems. Linear stability analysis of the physical system consisting of a vapor layer underlying a liquid layer of an inviscid fluid was carried out by Hsieh [3, 4]. He used the potential flow theory to solve the governing equations and observed that the heat and mass transfer phenomenon enhances the stability of the system if the vapor layer is hotter than the liquid layer. Ho [5] studied the problem of Rayleigh-Taylor instability taking heat and mass transfer into the analysis, but his study was restricted to the fluids of same kinematic viscosity. Adham-Khodaparast et al. [6] restudied the linear stability analysis of a liquid-vapor interface, but they considered liquid as viscous and motionless and vapor as inviscid moving with a horizontal velocity. Awasthi and Agrawal [7] extended the work of Hsieh [3] considering both fluids as viscous. The Kelvin-Helmholtz instability occurs when there is a relative motion between the fluid layers of different physical parameters. The study of heat and mass transfer on the Kelvin-Helmholtz instability of miscible fluids using viscous potential flow theory was made by Asthana and Agrawal [8]. Awasthi and Agrawal [9] studied the capillary instability when the fluids are miscible and viscous.

The presence of an electric field may change the fluid behaviour and its flow. The study of effects resulting from electric fields on fluid flows is called electrohydrodynamics (EHD). The impact of electric field on the stability of two fluid systems is one of the important problems in electohydrodynamics. The discontinuity of the electric properties of the fluids across the interface affects the force balance at the fluid-fluid interface, which may either stabilize or destabilize the interface in question. The study of the electrohydrodynamic Rayleigh-Taylor instability of two inviscid fluids in the presence of tangential electric field was considered by Eldabe [10]. He found that the tangential electric field has stabilizing effect. Mohamed et al. [11] studied the nonlinear electrohydrodynamic Rayleigh-Taylor instability of inviscid fluids with heat and mass transfer in presence of a tangential electric field and observed that heat and mass transfer has stabilizing effects in the nonlinear analysis. The effect of tangential electric field on the Rayleigh-Taylor instability when there is heat and mass transfer across the interface was studied by Awasthi and Agrawal [12].

In the VPF theory, we assume that the tangential part of viscous stresses is zero in case of free surface problems, but it is not possible in practical situations. To incorporate this discontinuity, Wang et al. [13] included an extra pressure term known as viscous pressure in the normal stress balance. Using the global energy balance, they found that this viscous pressure term will include the effect of tangential stresses. This theory is called viscous corrections for the viscous potential flow (VCVPF) theory. VCVPF analysis provides a new direction to deal with stability problems and it is getting attention of many researchers in recent times. Awasthi [14] applied VCVPF theory on the Rayleigh-Taylor instability of two viscous fluids when there is heat and mass transfer across the interface and observed that the irrotational shearing stresses stabilize the interface.

In view of the above investigations and keeping in mind the importance of electrohydrodynamics in a number of applications such as heat exchanger manufacturing [15], power generation, and other industrial processes, a study of the linear electrohydrodynamic Rayleigh-Taylor instability of the plane interface when there is heat and mass transfer across the interface is attempted. We use potential flow theory and the fluids are considered to be incompressible, viscous, and dielectric with different kinematic viscosities and permittivities, respectively, which have not been considered earlier. The effect of free surface charges at the interface is neglected. A dispersion relation that accounts for the growth of disturbance waves is derived and stability is discussed theoretically as well as numerically. A critical value of the electric field as well as the critical wave number is obtained. The effect of ratio of permittivity of two fluids on stability of the system is also studied and shown graphically. Various neutral curves are drawn to show the effect of various physical parameters such as electric field and heat transfer coefficient on the stability of the system.

## 2. Problem Formulation

**Figure 1:**
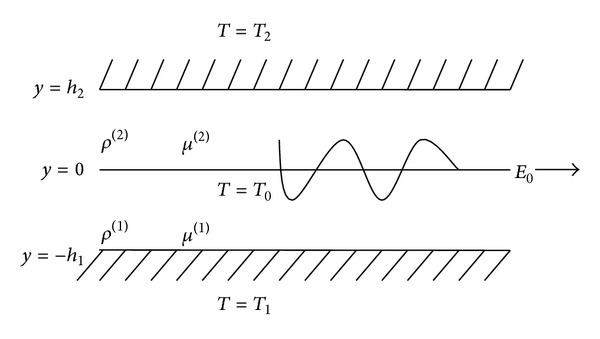
The equilibrium configuration of the system.

A system consisting of two incompressible, viscous, and dielectric fluid layers of finite thickness separated by a plane interface *y* = 0 is considered, as demonstrated in Figure 1. The lower fluid (1) occupies the lower region −*h*_1_ < *y* < 0, having thickness *h*_1_, density *ρ* ^(1)^, viscosity *μ* ^(1)^, and dielectric constants *ε* ^(1)^, and is bounded by the rigid plane surface *y* = −*h*_1_ while the upper fluid (2) occupies the outer region 0 < *y* < *h*_2_, having thickness *h*_2_, density *ρ* ^(2)^, viscosity *μ* ^(2)^, and dielectric constants *ε* ^(2)^, and is bounded by the rigid plane surface *y* = *h*_2_. The temperatures at *y* = −*h*_1_, *y* = 0, and *y* = *h*_2_ are taken as *T*_1_, *T*_0_, and *T*_2_, respectively. We assume that in the basic state, interface temperature *T*_0_ is equal to the saturation temperature because the fluids are in thermodynamic equilibrium. The external force at the interface is taken as the gravitational force *g* in the direction of (−*y*). In the present analysis, the fluids are taken as irrotational and incompressible.

To study the stability of the system, small disturbances are imposed on the equilibrium state. Then, the equation of the interface can be written as

(1) $$\begin{array}{c} F\left(x,y,t\right)=y-\eta \left(x,t\right)=0, \end{array}$$

where *η* represents the varicose interface displacement. The outward unit normal vector can be defined as

(2) $$\begin{array}{c} n=\frac{\nabla F}{\left|\nabla F\right|}={\left\{1+{\left(\frac{\partial \eta }{\partial x}\right)}^{2}\right\}}^{-1/2}\left(e_{y}-\frac{\partial \eta }{\partial x}e_{x}\right), \end{array}$$

where **e**_*x*_ and **e**_*y*_ are unit vectors along *x*- and *y*-directions, respectively.

Our analysis is based on the potential flow theory; therefore, velocity can be expressed as the gradient of the potential function; that is,

(3) $$\begin{array}{c} u_{j}=\nabla \phi^{\left(j\right)},\;\left(j=1,2\right). \end{array}$$

For incompressible fluids, the density is constant; the continuity equation takes the form

(4) $$\begin{array}{c} \nabla \cdot u_{j}=0. \end{array}$$

Combining (3) and (4), we have

(5) $$\begin{array}{c} \nabla^{2}\phi^{\left(j\right)}=0,\;j=1,2. \end{array}$$

In the present analysis, it is assumed that the two fluids are subjected to an external electric field *E*_0_, acting along *x*-axis and therefore

(6) $$\begin{array}{c} E_{j}=E_{0}e_{x}. \end{array}$$

We are assuming that the quasistatic approximation is valid; hence, the electric field can be written in terms of electric scalar potential function *ψ*(*x*, *y*, *t*) as

(7) $$\begin{array}{c} E_{j}=E_{0}e_{x}-\nabla \psi^{\left(j\right)},\;\left(j=1,2\right). \end{array}$$

Using Gauss's law, the electric potentials will satisfy Laplace's equation; that is,

(8) $$\begin{array}{c} \nabla^{2}\psi^{\left(j\right)}=0,\;\left(j=1,2\right). \end{array}$$

The normal component of velocity at the rigid surfaces *y* = −*h*_1_ and *y* = *h*_2_ should be zero; that is,

(9) $$\begin{array}{c} \frac{\partial \phi^{\left(j\right)}}{\partial y}=0\;\text{at}\;\;y={\left(-1\right)}^{j}h_{j},\;\;\left(j=1,2\right). \end{array}$$

The normal component of electric potential also vanishes at the rigid surfaces; that is,

(10) $$\begin{array}{c} \frac{\partial \psi^{\left(j\right)}}{\partial y}=0\;\text{at}\;\;y={\left(-1\right)}^{j}h_{j},\;\;\left(j=1,2\right). \end{array}$$

The tangential component of the electric field must be continuous across the interface; that is,

(11) $$\begin{array}{c} \left[E_{t}\right]=0, \end{array}$$

where *E*_*t*_( = |**n** × **E**|) is the tangential component of the electric field and [*x*] represents the difference in a quantity across the interface; it is defined as [*x*] = *x* ^(2)^ − *x* ^(1)^.

There is discontinuity in the normal current across the interface; charge accumulation within a material element is balanced by conduction from bulk fluid on either side of the surface. The boundary condition, corresponding to normal component of the electric field, at the interface is given by

(12) $$\begin{array}{c} \left[\varepsilon E_{n}\right]=0, \end{array}$$

where *E*_*n*_( = **n** · **E**) is the normal component of the electric field.

The interfacial condition, which expresses the conservation of mass across the interface, is given by the equation

(13) $$\begin{array}{c} \left[\rho \left(\frac{\partial F}{\partial t}+\nabla \phi \cdot \nabla F\right)\right]=0\;\text{at}\;\;y=\eta . \end{array}$$

In the present analysis, we have assumed that the amount of latent heat released depends mainly on the instantaneous position of the interface. Therefore, the interfacial condition for energy transfer is expressed as

(14) $$\begin{array}{c} L\rho^{\left(1\right)}\left(\frac{\partial F}{\partial t}+\nabla \phi^{\left(1\right)}\cdot \nabla F\right)=S\left(\eta \right)\;\text{at}\;y=\eta , \end{array}$$

where *L* is the latent heat released during phase transformation and *S*(*η*) denotes the net heat flux from the interface.

If *K*_1_ and *K*_2_ denote the heat conductivities of the two fluids, the heat fluxes in positive *y*-direction in the fluid phases 1 and 2 will be −*K*_1_(*T*_1_ − *T*_0_)/*h*_1_ and *K*_2_(*T*_0_ − *T*_2_)/*h*_2_, respectively. Therefore, the expression for net heat flux *S*(*η*) can be written as

(15) $$\begin{array}{c} S\left(y\right)=\frac{K_{2}\left(T_{0}-T_{2}\right)}{h_{2}-y}-\frac{K_{1}\left(T_{1}-T_{0}\right)}{h_{1}+y}. \end{array}$$

On expanding *S*(*η*) in the neighbourhood of *η* = 0, we have

(16) $$\begin{array}{c} S\left(\eta \right)=S\left(0\right)+\eta S^{\prime }\left(0\right)+\frac{1}{2}\eta^{2}S^{\prime \prime }\left(0\right)+\cdots . \end{array}$$

Since *S*(0) = 0 in the equilibrium condition, we obtain from (15)

(17) $$\begin{array}{c} \frac{K_{2}\left(T_{0}-T_{2}\right)}{h_{2}}=\frac{K_{1}\left(T_{1}-T_{0}\right)}{h_{1}}=G,\;\text{where}\;\;G\;\;\text{is}\;\;\text{a}\;\;\text{constant}. \end{array}$$

Since the fluids are miscible and there is heat and mass transfer across the interface, the interfacial condition for conservation of momentum will take the form

(18) ρ(1)(∇ϕ(1)·∇F)(∂F∂t+∇ϕ(1)·∇F)  =ρ(2)(∇ϕ(2)·∇F)(∂F∂t+∇ϕ(2)·∇F)   +(p2−p1−2μ(2)n·∇⊗∇ϕ(2)     ·n+2μ(1)n·∇⊗∇ϕ(1)     ·n−12[ε(En2−Et2)]+σ∇·n)|∇F|2,

where *p* is the pressure, *σ* is the surface tension coefficient, and **n** is the normal vector at the interface, respectively. Surface tension has been assumed to be a constant, neglecting its dependence on temperature.

## 3. Viscous Corrections for Viscous Potential Flow (VCVPF) Analysis

The viscous correction for the viscous potential flow analysis is another irrotational theory in which the shear stresses do not vanish. However, the shear stress in the energy balance can be calculated in the mean by the selection of an irrotational pressure which depends on viscosity.

Here, we have ignored the small deformation *η* in the linear analysis. Suppose that **n**_1_ = **e**_*y*_ denotes the unit outward normal at the interface for the lower fluid; **n**_2_ = −**n**_1_ is the unit outward normal for the upper fluid and **t** = **e**_*x*_ is the unit tangent vector. We will use the superscripts “*i*” for “irrotational” and “*v*” for “viscous” and subscripts “1” and “2” for lower and upper fluids, respectively. The normal and shear parts of the viscous stress will be represented by *τ* ^*n*^ and *τ* ^*s*^, respectively.

The mechanical energy equations for upper and lower fluids can be written as

(19) ddt∫V2ρ(2)2|u2|2dV =−∫Aρ(2)gηundA+∫A[u2·T·n2]dA  −∫V22μ(2)D2:D2dV =−∫Aρ(2)gηundA  −∫A[u2·n1(−p2i+τ2n)+u2·tτ2s]dV  −∫V22μ(2)D2:D2dV,

(20) ddt∫V1ρ(1)2|u1|2dV =−∫Aρ(1)gηundA+∫A[u1·T·n1]dA  −∫V22μ(1)D1:D1dV =−∫Aρ(1)gηundA  +∫A[u1·n1(−p1i+τ1n)+u1·tτ1s]dV  −∫V12μ(1)D1:D1dV,

where **D**_*j*_  (*j* = 1,2) denote the symmetric part of the rate of strain tensor for lower and upper fluids, respectively.

As the normal velocities are continuous at the interface, we have

(21) $$\begin{array}{c} u_{2}\cdot n_{1}=u_{1}\cdot n_{1}=u_{n}. \end{array}$$

The sum of (19) and (20) can be written as

(22) ddt∫V2ρ(2)2|u2|2dV+ddt∫V1ρ(1)2|u1|2dV =−∫Aρ(2)gηundA−∫Aρ(1)gηundA  −∫V22μ(2)D2:D2dV−∫V12μ(1)D1:D1dV  +∫A[un(−p1i+τ1n+p2i−τ2n)     + u2·tτ2s−u1·tτ1s]dA.

On introducing the two viscous pressure correction terms *p*_1_^*v*^ and *p*_2_^*v*^ for the lower and upper sides of the flow region, we can resolve the discontinuity of the shear stress and tangential velocity at the interface, so

(23) $$\begin{array}{c} \tau_{1}^{s}=\tau_{2}^{s}=\tau^{s},\;\;u_{2}\cdot t=u_{1}\cdot t=u_{s}. \end{array}$$

We assume that the boundary layer approximation has a negligible effect on the flow in the bulk liquid, but it changes the pressure and continuity conditions at the interface. Hence, (22) becomes

(24) ddt∫V2ρ(2)2|u2|2dV+ddt∫V1ρ(1)2|u1|2dV =−∫Aρ(2)gηundA−∫Aρ(1)gηundA  −∫V22μ(2)D2:D2dV−∫V12μ(1)D1:D1dV  +∫A[un(−p1i−p1v+τ1n+p2i+p2v−τ2n)]dA.

Now, we can obtain an equation which relates the pressure corrections to the uncompensated irrotational shear stresses by comparing (22) and (24):

(25) $$\begin{array}{c} \overset{}{\underset{A}{\int }}\left[u_{n}\left(-p_{1}^{v}+p_{2}^{v}\right)\right]dA=\overset{}{\underset{A}{\int }}\left[u_{2}\cdot t\tau_{2}^{s}-u_{1}\cdot t\tau_{1}^{s}\right]dA. \end{array}$$

It has been shown by Wang et al. [13] that in linearized problems, the governing equation for the pressure corrections is given by

(26) $$\begin{array}{c} \nabla^{2}p^{v}=0. \end{array}$$

Using the normal mode method, the solution of (20) can be written as

(27) $$\begin{array}{c} p_{1}^{v}=-\left(C_{k}\cosh ky+E_{k}\sinh ky\right)\exp\left[\left(ikx-i\omega t\right)\right],\\ p_{2}^{v}=-\left(D_{k}\cosh ky+F_{k}\sinh ky\right)\exp\left[\left(ikx-i\omega t\right)\right]. \end{array}$$

At the interface *y* = 0, the difference in the viscous pressure is expressed as

(28) $$\begin{array}{c} -p_{1}^{v}+p_{2}^{v}=\left[C_{k}-D_{k}\right]\exp\left(ikx-i\omega t\right). \end{array}$$

The equation of conservation of momentum (18) on including the viscous pressure can be written as

(29) ρ(1)(∇ϕ(1)·∇F)(∂F∂t+∇ϕ(1)·∇F) =ρ(2)(∇ϕ(2)·∇F)(∂F∂t+∇ϕ(2)·∇F)  +(p2i+p2v−p  1i−p1v−2μ(2)n·∇⊗∇ϕ(2)·n    +2μ(1)n·∇⊗∇ϕ(1)·n−12[ε(En2−Et2)]    + σ∇·n)|∇F|2.

Here, *p*_*j*_^*i*^ for (*j* = 1,2) is the irrotational pressure obtained by Bernoulli's equation.

## 4. Linearized Equations

The small disturbances are imposed on (11), (12), (13), (14), and (29) and retaining the linear terms, we can get the following equations:

(30) $$\begin{array}{c} \left[\frac{\partial \psi }{\partial x}\right]=0, \end{array}$$

(31) $$\begin{array}{c} \left[\varepsilon \left(E_{0}\frac{\partial \eta }{\partial x}+\frac{\partial \psi }{\partial y}\right)\right]=0, \end{array}$$

(32) $$\begin{array}{c} \left[\rho \left(\frac{\partial \phi }{\partial y}-\frac{\partial \eta }{\partial t}\right)\right]=0, \end{array}$$

(33) $$\begin{array}{c} \rho^{\left(1\right)}\left(\frac{\partial \phi^{\left(1\right)}}{\partial y}-\frac{\partial \eta }{\partial t}\right)=\alpha \eta , \end{array}$$

(34) $$\begin{array}{c} \left[\rho \left(\frac{\partial \phi }{\partial t}+g\eta \right)-p^{v}+2\mu \frac{\partial^{2}\phi }{\partial y^{2}}+\varepsilon E_{0}\frac{\partial \psi }{\partial x}\right]=-\sigma \frac{\partial^{2}\eta }{\partial x^{2}}, \end{array}$$

where *α* = *G*/*L*((1/*h*_1_)+(1/*h*_2_)).

The normal mode technique has been used to find the solution of the governing equations. We have considered the interface elevation in the form

(35) $$\begin{array}{c} \eta =C\exp\left(i\left(kz-\omega t\right)\right)+\text{c}.\text{c}., \end{array}$$

where *C* represents the amplitude of the surface wave, *k* denotes the real wave number, *ω* is the growth rate, and c.c. refers to the complex conjugate of the preceding term.

Now, using normal mode analysis and using the boundary conditions (30)–(33), the solution of (5) and (8) can be written as

(36) ϕ(1)=1k(αρ(1)−iω)Ccosh(k(y+h1))sinh(kh1) ×exp(ikx−iωt)+c.c.,ϕ(2)=−1k(αρ(2)−iω)Ccosh(k(y−h2))sinh(kh2) ×exp(ikx−iωt)+c.c.,ψ(1)=iE0(ε(2)−ε(1))(ε(1)tanhkh1+ε(2)tanhkh2) ×Ccoshk(y+h1)coshkh1exp(ikx−iωt)+c.c.,ψ(2)=iE0(ε(2)−ε(1))(ε(1)tanhkh1+ε(2)tanhkh2) ×Ccoshk(y−h2)coshkh2exp(ikx−iωt)+c.c.

The contribution of irrotational shearing stresses will be obtained by solving (25) along with (28). So, we have

(37) [Ck−Dk]=2kC[μ(1)(αρ(1)−iω)coth(kh1)    + μ(2)(αρ(2)−iω)coth(kh2)].

## 5. Dispersion Relation

We have used the expressions of *η*, *ϕ* ^(1)^, *ϕ* ^(2)^, *ψ* ^(1)^, *ψ* ^(2)^, and −*p*_1_^*v*^ + *p*_2_^*v*^ in (34) to find the dispersion relation which is a quadratic equation expressed as follows:

(38) $$\begin{array}{c} D\left(\omega ,k\right)=a_{0}\omega^{2}+ia_{1}\omega -a_{2}=0, \end{array}$$

where

(39)  a0=ρ(1)coth(kh1)+ρ(2)coth(kh2),a1=α(coth(kh1)+coth(kh2))+4k2(μ(1)coth(kh1)+μ(2)coth(kh2)),a2=(ρ(1)−ρ(2))gk+σk3+4k2α×(μ(1)ρ(1)coth(kh1)+μ(2)ρ(2)coth(kh2))+k2E02(ε(2)−ε(1))2(ε(1)tanh(kh2)+ε(2)tanh(kh2)).

For *E*_0_ = 0, (38) is reduced to dispersion relation as obtained by Awasthi [14]. In (38), putting *E*_0_ = 0 and neglecting the effect of irrotational shearing stresses, we get the dispersion relation as obtained by Awasthi and Agrawal [7].

If we use the transformation *ω* = *iω*_0_, the dispersion relation can be obtained in growth rate *ω*_0_ as

(40) $$\begin{array}{c} a_{0}\omega_{0}^{2}+a_{1}\omega_{0}+a_{2}=0. \end{array}$$

Now using the Routh-Hurwitz criteria [16] for (40), we get the stability conditions as follows:

(41) $$\begin{array}{c} a_{0}>0,\;a_{1}>0,\;a_{2}>0. \end{array}$$

If we use the properties of modified Bessel functions, *a*_0_ will always be positive. The viscosities are always positive and so *a*_1_ > 0. Therefore, the condition of stability reduces to *a*_2_ > 0; that is,

(42) (ρ(1)−ρ(2))gk+σk3+4k2α  ×(μ(1)ρ(1)coth(kh1)+μ(2)ρ(2)coth(kh2))  +k2E02(ε(2)−ε(1))2(ε(1)tanh(kh2)+ε(2)tanh(kh2))>0.

Hence, we conclude that the system is stable for *k* ≥ *k*_*c*_ and unstable for *k* < *k*_*c*_, where *k*_*c*_ is the critical value of the wave number.

Equation (42) can also be written as

(43) k2E02(ε(2)−ε(1))2(ε(1)tanh(kh2)+ε(2)tanh(kh2))  <(ρ(2)−ρ(1))gk−σk3−4k2α   ×(μ(1)ρ(1)coth(kh1)+μ(2)ρ(2)coth(kh2)).

From the above expression, it can be concluded that the system is stable for *E* ≤ *E*_*c*_ and unstable for *E* > *E*_*c*_, where *E*_*c*_ is the critical value of the electric field.

The condition for neutral stability can be written as

(44) (ρ(1)−ρ(2))gk+σk3+4k2α  ×(μ(1)ρ(1)coth(kh1)+μ(2)ρ(2)coth(kh2))  +k2E02(ε(2)−ε(1))2(ε(1)tanh(kh2)+ε(2)tanh(kh2))=0.

If the fluids are considered to be inviscid, that is, *μ* ^(1)^ = *μ* ^(2)^ = 0, heat and mass transfer has no effect on the stability criterion. Also, if there is no heat and mass transfer across the interface, that is, *α* = 0, the inviscid potential flow (IPF), VPF, and the VCVPF solutions predict the same critical wave number.

## 6. Dimensionless Form of Dispersion Relation

Let *h* = *h*_2_ + *h*_1_ be the characteristic length and $Q={\left[\left(1-\hat{\rho }\right)gh/\hat{\rho }\right]}^{1/2}$ represents the characteristic velocity. Then, the nondimensional forms of other parameters are defined as

(45) $$\begin{array}{c} \hat{k}=kh,\;\;\hat{\alpha }=\frac{\alpha h^{2}}{\mu^{\left(2\right)}},\;\;{\hat{h}}_{1}=\frac{h_{1}}{h}\equiv \varphi ,\\ {\hat{h}}_{2}=\frac{h_{2}}{h}=1-{\hat{h}}_{1},\;\;\hat{\rho }=\frac{\rho^{\left(1\right)}}{\rho^{\left(2\right)}},\;\;\hat{\mu }=\frac{\mu^{\left(1\right)}}{\mu^{\left(2\right)}},\\ \hat{\omega }=\frac{\omega_{0}h}{Q},\;\;\hat{\sigma }=\frac{\sigma }{\rho^{\left(2\right)}gh^{2}},\;\;\vartheta =\frac{\mu^{\left(2\right)}}{\rho^{\left(2\right)}hQ},\\ \hat{\varepsilon }=\frac{\varepsilon^{\left(1\right)}}{\varepsilon^{\left(2\right)}},\;\;{\hat{E}}^{2}=\frac{E^{2}\varepsilon^{\left(2\right)}}{\rho^{\left(2\right)}gh},\;\;\kappa =\frac{\hat{\mu }}{\hat{\rho }},\;\;\Lambda =\frac{\hat{\alpha }\vartheta^{2}}{\hat{\rho }}. \end{array}$$

Here, *φ* denotes the vapour fraction, *κ* represents the kinematic viscosity ratio, and Λ denotes the alternative heat transfer coefficient.

The dimensionless form of (40) can be written as

(46) [ρ^coth(k^h^1)+coth(k^h^2)]ω^2  +ϑ[α^(coth(k^h^1)+coth(k^h^2))    +4k^2(μ^coth(k^h^1)+coth(k^h^2))]ω^  −[ρ^k^{1+σ^k^2(ρ^−1)+k^E^2(ρ^−1)      ×(ε^−1)2(ε^tanh(kh1)+tanh(kh2))}   −4k^2α^ϑ2{κcoth(k^h^1)+coth(k^h^2)}]=0,

and non-dimensional form of (44) is given by

(47) 1+σ^k^2(ρ^−1)+k^E^2(ρ^−1)(ε^−1)2(ε^tanh(kh1)+tanh(kh2)) −4k^Λ{κcoth(k^h^1)+coth(k^h^2)}=0.

## 7. Results and Discussions

In this section, we have carried out the numerical computation using the expressions presented in the previous sections for a film boiling condition. We have taken vapour and water as working fluids identified with phase 1 and phase 2, respectively, such that *T*_1_ > *T*_0_ > *T*_2_. We are treating steam as incompressible since the Mach number is expected to be small. The water-vapour interface is in saturation condition in film boiling situation and the temperature *T*_0_ is equal to the saturation temperature. We have considered the following parametric values for the analysis:

(48) $$\begin{array}{c} \rho^{\left(1\right)}=0.001\;\text{gm/}{\text{cm}}^{3},\;\;\rho^{\left(2\right)}=1.0\;\text{gm/}{\text{cm}}^{3},\\ \mu^{\left(1\right)}=0.00001\;\;\text{poise},\;\;\mu^{\left(2\right)}=0.01\;\;\text{poise},\\ \sigma =72.3\;\text{dyne/cm}. \end{array}$$

Since the transfer of mass across the interface represents a transformation of the fluid from one phase to another, there is regularly a latent heat associated with phase change. It is basically through this interfacial coupling between the mass transfer and the release of latent heat that the motion of fluids is influenced by the thermal effects. Therefore, when there is mass transfer across the interface, the transformation of heat in the fluid has to be taken into the account. Neutral curves for wave number divide the plane into a stable region above the curve and an unstable region below the curve while neutral curves for the electric field divide the plane into a stable region below the curve and an unstable region above the curve.

**Figure 2:**
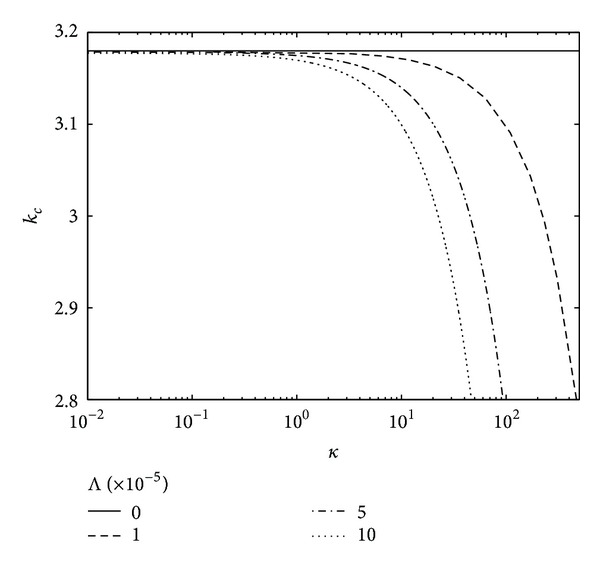
Neutral curves for critical wave number when $\hat{E}=1$, *φ* = 0.1 for the different values of heat transfer coefficient Λ.

The effect of alternative heat-transfer capillary dimensionless group Λ on the neutral curves for critical wave number has been shown in Figure 2 when the electric field intensity $\hat{E}=1$. Here, we have found that if we Λ constant and increase *κ*, the critical wave number *k*_*c*_ reduces for fixed value of vapor fraction *φ*; hence, the VCVPF theory predicts longer stable waves. As alternative heat-transfer capillary dimensionless group Λ increases, the stable region also increases. As Λ increases, the stable region also increases. The coefficient Λ is directly proportional to the heat flux and therefore, heat flux has stabilizing effect on the system. This is the same result as the one obtained by Awasthi [14] for the Rayleigh-Taylor instability with heat and mass transfer in the absence of electric field. Therefore, we state that the behaviour of heat flux is not affected by the presence of an electric field. We can explain the effect of heat and mass transfer on the stability of the system taking local evaporation and condensation at the interface. Crests are warmer at the perturbed interface because they are closer to the hotter boundary on the vapour side; thus, local evaporation takes place, whereas troughs are cooler and thus condensation takes place. The liquid is protruding to a hotter region and the evaporation will diminish the growth of disturbance waves.

**Figure 3:**
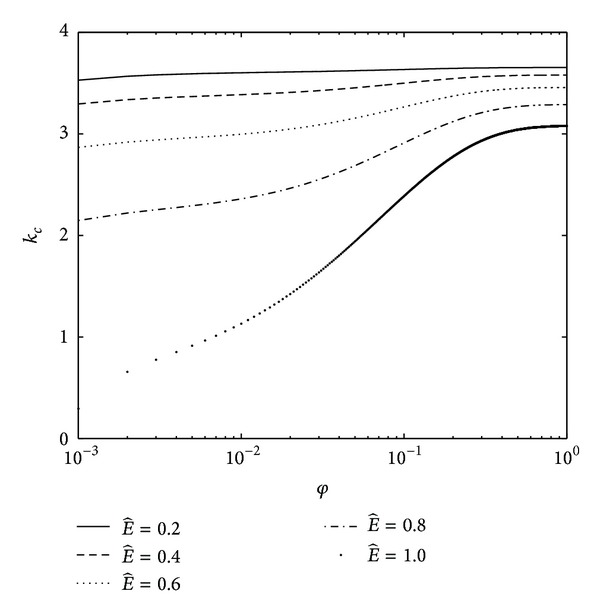
Neutral curves for critical wave number when Λ = 10^−5^ for the different values of electric field intensity $\hat{E}$.

The effect of electric field intensity $\hat{E}$ on the neutral curves for the critical wave number *k*_*c*_ is illustrated in Figure 3. We observe that for a fixed value of *κ* and Λ, the critical wave number *k*_*c*_ decreases on increasing electric field intensity $\hat{E}$. Therefore, it is concluded that $\hat{E}$ has stabilizing effect. If electric field is present in the analysis, the term contributed from the applied electric field is added in the left-hand side of (47) so that critical value of wave number decreases and system will become more stable. The concept of polarization can explain the physical mechanism of this phenomenon. The polarization forces due to differences in permittivities and perturbed velocities have the effect of pushing the disturbance waves and therefore, electric field stabilizes the interface. It is also observed from Figure 3 that as vapour thickness increases, the stable region decreases and so vapour thickness plays a destabilizing role. On increasing the vapor fraction, more evaporation takes place at the crests. This additional evaporation will increase the amplitude of the disturbance waves and the system becomes destabilized.

**Figure 4:**
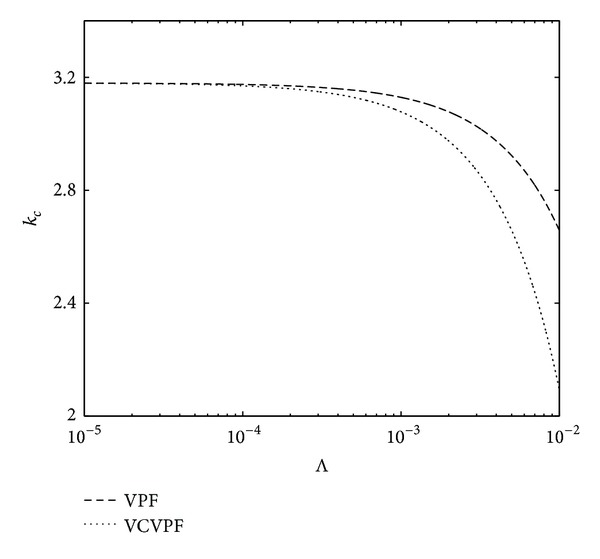
Comparison between the neutral curves for critical wave number obtained for VPF as well as VCVPF analysis when $\hat{E}=1.0$.

In Figure 4, the effects of irrotational viscous pressure on the Rayleigh-Taylor instability with heat and mass transfer have been studied. Here, a comparison is performed between the neutral curves of wave number *k*_*c*_ obtained from the present analysis (VCVPF solution) and those obtained from the VPF solution when the electric field $\hat{E}=1$. We observe that as the values of heat transfer coefficient increase, the stable region increases in the VCVPF solution in comparison with the VPF solution; this indicates that the effect of irrotational viscous pressure stabilizes the system in the presence of heat and mass transfer.

**Figure 5:**
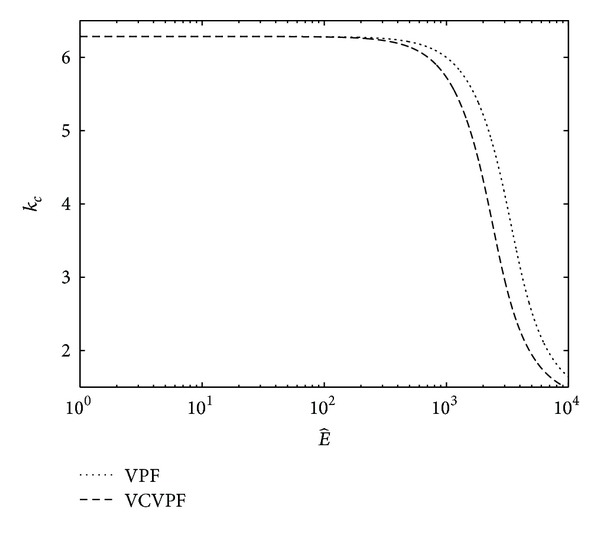
Comparison between the neutral curves for critical wave number obtained for VPF as well as VCVPF analysis when Λ = 10^−5^.

Figure 5 shows the comparison between the neutral curves of wave number obtained by the VPF analysis and those obtained by VCVPF (present) analysis for different electric fields. As its intensity increases, the critical wave number decreases for both VPF and VCVPF analyses; however, in case of VCVPF solution it decreases faster, Hence, at the higher values of electric field, VCVPF solution is more stable than VPF solution.

## 8. Conclusion

The effect of tangential electric field on the Rayleigh-Taylor instability is studied when there is heat and mass transfer across the interface. The viscous correction for viscous potential flow theory is used for investigation. The dispersion relation is obtained, which is a quadratic equation in growth rate. The stability condition is obtained by applying Routh-Hurwitz criterion. A critical value of electric field as well as critical wave number is obtained. The system is unstable when the electric field is greater than the critical value of electric field; otherwise, it is stable. It is observed that the heat and mass transfer has stabilizing effect on the stability of the system and this effect is enhanced in the presence of an electric field. The heat and mass transfer completely stabilizes the interface against capillary effects even in the presence of an electric field. It is also observed that the tangential electric field increases the stability of the system. The VCVPF solution is more stable than the VPF solution at the high electric field intensity as well as high heat transfer.
